# The intelligence paradox; will ET get the metabolic syndrome? Lessons from and for Earth

**DOI:** 10.1186/1743-7075-11-34

**Published:** 2014-07-29

**Authors:** Alistair V W Nunn, Geoffrey W Guy, Jimmy D Bell

**Affiliations:** 1 School of Pharmacy, University of Reading, Whiteknights, Reading, Berks RG6 6AP, UK; 2 GW pharmaceuticals, Porton Down, Salisbury, Wiltshire SP4 0JQ, UK; 3 Metabolic and Molecular Imaging Group, MRC Clinical Sciences Centre, Imperial College London, Hammersmith Hospital, London W12 0NN, UK

**Keywords:** Intelligence, Obesity, Hormesis, Exercise, Metabolic syndrome, Type 2 diabetes, Environment, Aging, Mitochondria, Proton gradients, Evolution, Fermi paradox, Entropy

## Abstract

Mankind is facing an unprecedented health challenge in the current pandemic of obesity and diabetes. We propose that this is the inevitable (and predictable) consequence of the evolution of intelligence, which itself could be an expression of life being an information system driven by entropy. Because of its ability to make life more adaptable and robust, intelligence evolved as an efficient adaptive response to the stresses arising from an ever-changing environment. These adaptive responses are encapsulated by the epiphenomena of “hormesis”, a phenomenon we believe to be central to the evolution of intelligence and essential for the maintenance of optimal physiological function and health. Thus, as intelligence evolved, it would eventually reach a cognitive level with the ability to control its environment through technology and have the ability remove all stressors. In effect, it would act to remove the very hormetic factors that had driven its evolution. Mankind may have reached this point, creating an environmental utopia that has reduced the very stimuli necessary for optimal health and the evolution of intelligence – “the intelligence paradox”. One of the hallmarks of this paradox is of course the rising incidence in obesity, diabetes and the metabolic syndrome. This leads to the conclusion that wherever life evolves, here on earth or in another part of the galaxy, the “intelligence paradox” would be the inevitable side-effect of the evolution of intelligence. ET may not need to just “phone home” but may also need to “phone the local gym”. This suggests another possible reason to explain Fermi’s paradox; Enrico Fermi, the famous physicist, suggested in the 1950s that if extra-terrestrial intelligence was so prevalent, which was a common belief at the time, then where was it? Our suggestion is that if advanced life has got going elsewhere in our galaxy, it can’t afford to explore the galaxy because it has to pay its healthcare costs.

## Introduction

This paper outlines a simple concept that may help to explain why humanity, and perhaps any other advanced civilisations across the galaxy, would experience a reduction in healthy life expectancy and possibly a cognitive plateau as they develop technology that would impact their ability to travel beyond their own star system. The concept is rooted in the evolution of intelligence, which in its broadest definition is an energy seeking and maintenance mechanism that arose to improve adaption to a constantly challenging environment by capturing information. This whole process may be driven by hormesis and entropy, which may be fundamental to the formation and evolution of life as a self-ordered system that accelerates the flow of energy throughout the universe by utilising free energy gradients.

Hormesis can be thought of an epiphenomenon that describes the biological adaptive response to an ever-changing environment [1]. It could well be a universal principle and applicable to life any anywhere in the universe, and could help answer one of the key questions for humanity: are we alone in our galaxy? Given the age of our star (young) and the sheer number of other stars and potentially life supporting planets, it seems unlikely that we are alone, especially if entropy and the nature of the universe biases towards the formation of life. Latest estimates suggest that at least in 100 million planets in our galaxy could support complex life [2] – especially if one considers that it seems to exist in more hostile environments than previously thought, and may possibility not even require the same chemistry [3]. However, if this is the case, as the famous Italian physicist Enrico Fermi asked so many years ago: “where is it”, and “why haven’t we been contacted by it yet?” This has come to be known as the “*Fermi paradox*”.

The question of whether we are alone is one that has been posed by many great minds throughout human history. However, the first serious attempts to answer this question were only really possible with the advent of modern science. It was Frank Drake who used his now famous equation to estimate the probability of life in our galaxy, which in turn led to the first serious attempts using radio telescopes to listen out for signs of extra-terrestrial life (ET). The debate still goes on, but one interesting conclusion has been is that if there is advanced life, probably the best way for it to determine if there is other life in the galaxy is to go looking for it, so colonising it in the process [4].

We therefore argue that, accepting the uncertainties about making a precise prediction about whether or not other non-human advanced civilisations could have evolved, if such a civilisation did reach a certain technological level they would remove, like we have, all environmental stresses. In fact, life probably does not have to reach an advanced level to start to remove these stresses, as it seems many forms of earthly life can alter their environment, and in many instances, this seems to be adaptive process to help them survive. For example, many trees shed leaves/needles that inhibit the growth of other plants around them, beavers build dams and termites construct huge nests. Thus humans are not alone in altering their environment to make their existence easier; indeed, this may, as we suggest later, be an inevitable consequence of intelligence.

However, if life does get advanced enough on another planet, will it also develop space travel? As we have no other comparison, we will have to assume, as humans have demonstrated, that the ability to greatly change the environment using machine technology will eventually lead to the ability to leave the surface of a planet. So what might put a break on reaching inter-stellar travel? Developments at NASA suggest it is not technology [5]. Our suggestion is that the development of a comfortable environment, wherever it occurs, could lead to similar physiological dysfunctions as we observe across the current human population. It is possible that ET may be suffering from the same obesity-related conditions that affect humanity today*.*

It could therefore be the cost of healthcare that prevents advanced space travel. But this may not be the only reason; inherent in the stress-induced information capture property of life is what might happen if this stress is removed. Diseases associated with calorie excess and a sedentary lifestyle, such as the metabolic syndrome, can be associated with reduced cognition [6,7]. One link may be via inflammation that can suppress cognition [8] and at a more basic level, mitochondrial function [9]. In fact, mitochondria are essential for brain function [10]. Thus, it is likely that as hormesis is key in stimulating mitochondrial biogenesis [11], it can reverse many of the negative effects of a poor lifestyle [12] – up to and including any potential reduction in cognition. Although we do not know if mitochondria would be part of alien life, it is possible that oxygen might be an important electron acceptor on other planets. Hormetic stimulation of an electron transport chain (or perhaps an alien equivalent), where ever it may occur, may thus not only have been very important in the evolution of intelligence, but also its maintenance. Removing it may thus affect intelligence.

If this is the case, is there a deeper and more fundamental explanation for the relationship between mitochondrial function and intelligence, and thus, lifestyle? Can it be understood using perhaps the most fundamental branch of science attempting to explain the universe, quantum theory? Hameroff and Penrose have suggested a quantum explanation of consciousness involving microtubules [13]; we suggest that we might be able to take this a step further using the theories of Froehlich and mitochondrial electric fields [14] to provide an explanation why mitohormesis is essential to maintain intelligence. An important clue to this might come from the possibility that a poor lifestyle might induce inflammation because without the necessary stress (hormesis) the biological system, which is essentially an incredibly complex self-ordered system created in response to a tough environment, may start to suffer instability. To put it simply, life evolved, and now maintains its order, in response to a constantly changing and challenging environment. Remove the stress and life begins to lose its order, resulting in spiralling oxidative stress.

Combining these thoughts, we set out to demonstrate that at least part of the explanation of the Fermi Paradox is an intelligence paradox whereby advanced civilisations essentially become very comfortable and remove all stressors, which leads to spiralling health care costs and potentially, either a plateau in average intelligence, or even a fall. Clearly, neither of these would support the development of interstellar space travel. The lessons from and for Earth are therefore obvious.

## Setting the scene: life, intelligence and hormesis

In order to help explain our hypothesis, it is helpful to define some well-known terms that many people use regularly; it is also important to understand some of the latest thinking on what life is, what it depends on and how it might have arisen. In particular, the link between stress, energy generation, and information capture, which might hint at the most basic reason as to why life exists at all.

### Origins of life

Although we do not know with absolute certainty, data is beginning to suggest that life could be an inevitable consequence of energy gradients, where entropy may have driven local order by utilising “outside” energy within a self-generating enclosed system. With regards to life on earth, this appears to be related to the ability to utilise proton gradients to drive endergonic cellular processes by exploiting the Gibbs free energy, which is contained in thermodynamic disequilibrium between ATP and ADP. As this disequilibrium is being constantly used up for maintaining the entropy decrease, an “outside” energy source must be tapped into to keep ATP/ADP ratios high. One possible origin for life may therefore have been around deep sea alkaline thermal vents, where proton gradients and reduction-oxidation (redox) reactions drove its development [15-18].

This gradient is echoed today in the modern cell, most obviously in the mitochondrion that maintains extremely high potentials across its membranes – equivalent to a bolt of lightning, which is about 30 million volts per metre and is vital for powering active transport across membranes, as well as the evolution of multicellular life. Any collapse in this membrane potential rapidly leads to free-radical leak and programmed cell death, and explains why mitochondria still retain 13 genes essential for oxidative phosphorylation, as it enables them to respond individually (reviewed by Lane, 2010) [19]. Thus maintaining this potential is vital to all modern complex life, demonstrating our complete dependency on proton gradients.

### Life, information and intelligence

Information is a pivotal property of life that enables it to adapt to its environment. Indeed, it has been suggested that the emergence of life may have corresponded to a physical transition associated with a shift in the causal structure, where information gained direct and context-dependent causal efficacy over the matter in which it is instantiated – an algorithmic origin of life [20].

This would suggest that all life, to some degree, displays a form of “intelligence”. For instance, it might include plants, which display an ability to form an “inner representation” or “cognitive map” of their environment and thus fulfil some of David Stenhouse’s original definitions in relation to the evolution of intelligence [21]. Another way to view this is that life has to have an awareness of “past and future” in order to adapt, and thus displays “chronognosis”; as life became more complex, this awareness of time extended – in bacteria it may be very short, but it reaches a pinnacle in humans [22] . But perhaps one of the most interesting ideas in relation to this is the quantum theory of consciousness, as proposed by Hameroff and Penrose, whereby consciousness can be explained by an orchestrated coherent quantum process in microtubules and the objective reduction of a quantum state; this therefore links a basic property of the universe with life and awareness itself [13]. Although these ideas are still controversial, it does suggest that the ability to adapt is dependent on acquiring and using information, and this is linked to a fundamental property of the universe where life is perhaps an inevitable property of this system. In effect, awareness of an environment may only be fleeting for a primitive organism, but becomes continuous as complexity evolves.

To fully understand this information system, the traditional concept of cellular data storage and evolution needs to be looked at in a new light. For many years the concept of evolution by natural selection has been broadly accepted, even though at the time the basic units of inheritance had not been fully identified. With the discovery of the double helix and DNA, and thus a biochemical description of the molecules required for genes, many thought that we had finally got to the bottom of the inheritance system. However, once again new data has overturned this; it appears that although genes are part of the information storage system, they are only one part. In fact, the gene-centric view of neo-Darwinism, or the modern synthesis, is now being challenged by our rediscovery of epigenetics. It is becoming clear that the entire cell is a network of information systems, which enables the instant capture of environmental information that can be passed on without alterations in the basic DNA code. In the words of the British biologist Denis Noble: “*acquired characteristics can be inherited, selection is multilevel, the genome is just an organ in the cell and genomes are not isolated from the environment*” [23].

The implication behind this is critical to our understanding of how the environment modulates life; it is actually far more plastic than previously thought. It would explain why “intelligence” is fundamental to life and why evolution would constantly act to improve it. All in all, this supports the concept that higher order intelligence evolved from lower order intelligence [24]. Further proof of this may well be the increasing levels of complexity now being discovered in relation to DNA transcription; it is turning out that the so called “junk DNA” is actually far more important than was previously realised. Latest estimates suggest that in humans, as much as 84% of DNA is transcribed, and since protein coding genes comprise less than 3%, there is much we still do not know, including the ever increasing numbers of what appear to be functional long intergenic noncoding RNAs (lincRNA) [25].

### Homeostasis, homeodynamics and hormesis

For most of evolution life has lived under varying degrees of stress arising from the environment. Stress, in this context, can be defined as *“that which induces perturbation of internal systems from normal”*. Hence the term “homeostasis” has been used to describe the way an organism keeps it systems operating within a normal range – a term that is still being adapted to include newer concepts, such as behavioural pre-emptive control [26]. However, more recently it has been suggested that a more appropriate term would be “homeodynamic”, which recognises the highly dynamic state of life as it constantly interacts with, and adapts to, its environment; in effect it is stable because it is dynamic [27,28]. This adaptive process, which is driven by stress, is encapsulated by the concept of “hormesis”. In the fields of biology and medicine hormesis is defined as “*an adaptive response of cells and organisms to a moderate (usually intermittent) stress*” [29]. Critically, hormesis can strengthen homeodynamics, in particular, by upregulating the stress response and thereby improving maintenance and repair mechanisms [30]. This has led to the concept that ageing is related to the loss of “homeodynamic space” and the reducing ability of an organism to adapt to perturbations in both internal and external environments [31].

## The hypotheses

### Hormesis is essential for complex biological systems

The fundamental principle of biological systems, which are based on chemical reactions and kinetics and subject to basic physical laws, is that they are inherently non-linear dynamically unstable, but show emergent self-organisational properties under perturbation. In other words they can only exhibit the appearance of stability by negative regulation. It is this very instability that has enabled evolution to occur [32,33].

One such emergent property appears to be “swarm intelligence” [34], defined as the collective behaviour of decentralized, self-organized systems. In relation to biological systems, this self-organising emergent property appears to arise in response to environmental perturbation and gives rise to the idea of “robustness” [35]. This would suggest that life would not have developed at all, let alone evolved intelligence, in a stable setting.

In simple terms, the emergence of life and intelligence, and thus adaptability, are products of constantly stressing a complex set of molecules. This would explain why hormesis, which embraces the adaptive response to non-lethal stress, increases homeodynamic robustness. It would also explain why removing stress leads to destabilisation of the system. In effect, hormesis is essential to maintain optimal fitness of a complex biological system, because without it, it becomes unstable. A level of stress is essential to induce self-organising emergent properties and maintain optimal function.

### Hormesis underpins the evolution of intelligence

Change is information which requires energy; therefore there is a direct relationship between maintaining energy production and information. If energy production is not challenged, then the need for information storage and therefore sophisticated mechanisms of adaptation become redundant. On the other hand, if energy production is continually challenged by environmental stresses, natural selection would not only select for the means to protect it, but would have evolved a system that could maximise the capture and processing of information to ensure maximum adaptability, in other words “intelligence”.

At the core of energy production is of course the proton gradient, which in higher order biosystems is mainly found in the mitochondria. Thus, if mitochondrial function is continuously compromised, the resulting increase in oxidative stress is used to promote upregulation of anti-oxidant systems that can preserve cellular function, as well as modulating mitochondrial dynamics [11]. In effect, this preserves and enhances the ability to capture information. From this, it can be theorised that hormesis must have played a pivotal role in the development of intelligence.

### Mitochondria are key to increased intelligence and longevity - SAPIENT

It has been proposed that mitochondrion enabled primitive cells to generate energy levels capable of supporting much greater complexity; in effect, more genes meant more proteins [19]. Memory, or the storage of information, is certainly an attribute of most living species, although where the boundary of self-awareness actually lies beyond humans, is still debated [36]. However, multiple systems display “cognition”, which is the ability to compare an internal model with an external one and respond to it, and in simple terms, consciousness may be the product of the integration of many such systems via the “global work space” concept. The whole system is highly tuneable, and suggests an underlying mechanism to explain recognisable “conscious” behaviours common to many species [37].

Another way of looking at this is that mitochondria enabled much more information to be captured from the environment, facilitating a real-time virtual internal model – an energy-dependent molecular memory that allowed the system to constantly compare and adjust for differences in the environment. This has led us to the concept of *Soma AdaPtabIlity Encoded iNformaTion* (SAPIENT), where increasing complexity enables more “soft-wired” information to be stored, as opposed to “hard-wired” in DNA. This would result in increased dynamic stability due to greater system redundancy. A critical outcome of this is that it would enhance the survival of the individual as a means of species promulgation. In contrast, although single celled life can display considerable individual adaptability, they are more reliant on rapid proliferation and classical natural selection to enable the species to adapt. This would suggest that evolution of longevity is a product of increasing complexity and soma memory, and in effect, life became less reliant on traditional natural selection for adaptability.

Thus an important outcome of the increased energy supply and intelligence is the evolution of an increased lifespan for many species, as they could adapt without resorting to natural selection by DNA mutation. It would also explain the emergence of consciousness as a kind of meta-cognition to enhance this adaptability as it enabled a greater ability to have an awareness of the future and develop behavioural pre-emptive mode of actions to maximise survival.

### The biphasic effects of proton gradient stress, longevity and SAPIENT

As we have suggested, the proton gradient plays a key role in adaptability and thus, longevity. As oxidative phosphorylation is the most efficient way to produce energy, but is associated with increased free radical production, then a cell might couple reliance on oxidative phosphorylation with increased anti-oxidant production to ensure this information system is sustained to optimise the individual’s survival. This is particularly important when energy supplies are limited. However, if the proton gradient is degraded irreparably, survival of a species will become more dependent on classical natural selection as the soft encoded information degrades and this ability will be lost. Hence, the ability to modulate functional longevity of the individual in relation to stress might also be a consequence of this to optimise survival of the species. It is therefore possible to make a prediction based on this principle: functional longevity will follow a “U” shaped curve. No stress will result in intermediate longevity, a small amount of stress will increase it, while too much will reduce it and force genetic adaptation.

In fact the above prediction has been long been observed. Natural selection has resulted in a plethora of different solutions for the survival of a species, ranging from short-lived organisms with very high reproductive rates, to very long lived organisms with very low reproductive rates. It is also clear that organisms also have adapted to a constantly changing energy supply and demand, and thus have to balance investment in reproduction against individual longevity. These observations have led, through several theories of aging [38], to the “disposal soma” theory by Kirkwood and Holliday – which may be applicable to many higher animals and their lifespans [39].

The disposal soma idea basically suggests that longevity, and the effective rate of ageing, is determined by a trade-off between energy requirements for reproduction, and that required to maintain the macromolecular milieu, including DNA, in a state that enables cells to function effectively. Not only can this be applied to evolution of absolute lifespan, but also to individuals to fine tune their functional longevity in times of hardship. In effect, in times of hardship, energy is diverted to somatic maintenance, and away from reproduction – enabling the animal to breed again another day [40].

To build on the theory of Kirkwood and Holliday, it can therefore be said that somatic maintenance is critical to maintaining fidelity of information, and thus, ensuring adaptability. Hence, when we move away from a gene-centric view of life, where cells are simply carriers of genes, to an integrated view, it becomes clear that all systems must be maintained to ensure fidelity for the cell to maintain its homeodynamic space and robustness. At a higher level, the survival of a species will depend on integrating signals from the proton gradient to adjust individual longevity.

In effect, when energy is available, there is less need to defend the proton gradient, as reproduction can occur. But as soon as it becomes limiting, it has to be protected to enable the individual to survive, which ties in with improving intelligence. Hence, somatic maintenance enhances SAPIENT to maintain adaptability, so ensuring that the proton gradient is protected.

### Quantum SAPIENT

One of the inherent properties of life is its awareness of time, as entropy can only go in one direction and can never be reversed. In fact, chronognosis, or biological time awareness, can be viewed as an inherent property of intelligence, thus, natural selection would have favoured it is a survival trait [22]. Hence advanced development of time awareness may have arisen due to the incorporation into primitive cells of the mitochondrion, which allowed greater complexity. Can we link the SAPIENT idea to this? Potentially we can, especially if we embrace the idea of the role of electromagnetic fields in living organisms (reviewed by Pokorny, [41]).

Classical theory suggests that consciousness is a product of synaptic complexity and although it could explain complex behaviour, it does not actually explain “awareness”. One possible explanation of this is the quantum theory of consciousness by Hameroff and Penrose involving microtubules, where tubulin can essentially act as a kind of quantum computer and lead to an orchestrated objective reduction, where the ability of large numbers of cells (neurons) to maintain quantum coherence, which might be perceived as consciousness, is achieved by quantum tunnelling at gap junctions [13]. This idea is linked to the biological electric field ideas of Fröhlich (reviewed by Srobar, 2012 [14]), who suggested that there is coupling of the mitochondrial electric field with dipole containing proteins, such as microtubules and the possibility of resonant energy transfer between the two. Importantly, the mitochondrial electric field also induces order in the water around the mitochondrion, which has the effect of damping microtubule oscillations. Possible support for this idea is suggested by the observation that single microtubules demonstrate enhanced electrical conduction at warm temperatures, and behave as multi-level memory switches, akin to modern computer flash memory chips [42,43]. It has also been proposed that biophotons produced by mitochondria might, theoretically, influence the quantum states of microtubules, and thus potentially, neuronal signalling [44]. Thus, it could be said that the ability of the microtubule system to maintain an orchestrated objective reduced state across the brain, and thus consciousness, is totally dependent on mitochondrial function.

Although conventional biochemistry can also explain why failure of mitochondrial function could explain a loss of consciousness, it is interesting that anaesthetics have been shown to not only inhibit microtubule function [45], but critically their potency is known to relate to their partition coefficient in lipid membranes, which has led to the hypothesis that they may work, in part, by depressing the transition melting point and thus altering free energy of membranes. This latter point has led to some authors proposing a thermodynamic explanation of general anaesthesia [46]. However, as might be predicted from the lipid effects, they also disrupt mitochondrial function and have been shown, at appropriate concentrations, to act as preconditioning agents [47,48]. Thus not only do they have the potential to have a mitohormetic effect, they can also modulate both membranes and the microtubule system. In effect, anaesthetics at a high enough concentration not only disrupt membrane thermodynamics, but also mitochondrial and microtubule function, which would rapidly lead to quantum decoherence and loss of consciousness. But, at low concentrations, they might induce adaptation.

Another important relationship between mitochondria and microtubules is calcium, which plays a critical role in microtubule stabilisation and its electrical transfer properties [49,50]. For instance, calcium uptake into the cell stimulates mitochondrial function, so enhancing energy production, as well as ROS. But as calcium levels increase, mitochondria can buffer it, and energy production becomes inhibited. They then release it back into the cytosol, where it is pumped out of the cell, so maintaining a strong extracellular-intracellular calcium gradient. If they take up too much calcium, then they can initiate apoptosis. But perhaps one of their most interesting properties is that under mild stress, such as reduced glucose or oxygen, they can also form a network via fusion, which enhances cell signalling; as stress increases, they then undergo fission, which breaks up the signalling network – so effectively invoking a negative feedback mechanism [51]. It is also thought that a fused network of mitochondria can be electrically united and transfer energy [52]. Hence, mild stress seems to invoke improved electrical conduction through the cell and potentially, enhance quantum coherence. Of particular relevance here is that the cytoskeleton and the mitochondrial system is closely linked, and in particular, plays a very important role at the neuronal synapse [53]. Thus, there may be a direct link to mild stress and an enhanced ability to store and transmit information.

In summary, although mitochondrial function is key in supplying ATP, and thus, could be seen to be essential in conventional models of brain function, there is also a strong precedent to suggest that quantum field effects may well also be important. One of the most interesting effects here could be synaptic protection to ensure quantum tunnelling and thus, quantum coherence by maintaining high field strengths. Mildly stressing cells, for instance, by a sudden calcium influx can enhance mitochondrial potential; even if mitochondrial function then decreases, rebound mitochondrial biogenesis and anti-oxidant capacity would then enhance the ability of the cell to store information. Thus anything that challenges the proton gradient would thus, potentially, enhance the ability of the cell to capture information: Q-SAPIENT.

### A lack of hormesis leads to system instability and mitochondrial dysfunction

As we have mentioned previously stress is an important part of ensuring that inherently dynamically unstable biochemical networks can be stabilised, which suggests that without stress, they might diverge from this stability as the impetus for emergent self-organisation is removed. In this system, this divergence might appear as reduced ability to managed redox, which in turn, would stimulate inflammation – and potentially lead to a vicious cycle as the system becomes more unstable. Management of redox is therefore key; we have previously suggested via the concept of “redox-thriftiness” that hormesis fine tunes insulin resistance via regulation of mitochondrial function and anti-oxidant pathways [12]: James D Watson, the Nobel laureate, has also recently emphasised the point that type 2 diabetes can be viewed as a redox disease [54].

Empirical evidence does appear to indicate that a shift towards mitochondrial production of energy, and away from glycolysis, can be induced by “pseudo-starvation” and activation of AMPK, an important detector of energy status; this process is now thought to be anti-inflammatory. In contrast, inflammation induces a shift towards glycolysis and utilises the mitochondrion to produce ROS for microbial killing, but provides ATP to maintain mitochondrial membrane potential, which helps prevent apoptosis. This is reflected in inflammatory cells being more reliant on glycolysis and anti-inflammatory cells being more reliant on oxidative phosphorylation. This also mirrors growth being associated more with glycolysis, and oxidative phosphorylation with non-growth. In effect, calorie restriction suppresses the immune system, while calorie excess activates it [55]. During sepsis, mitochondrial function is suppressed and appears to result in increased autophagy [56-58]. Hence, upregulation of mitophagy, increased mitochondrial biogenesis and antioxidant systems are key in counter-acting inflammatory damage and maintaining mitochondrial function [59]. In this way mitochondria, as well as providing energy for metabolism, are also key in controlling inflammation and its resolution [60].

This is thus another way of looking at one of the potential problems of living in an environment without hormesis. As the system destabilises, inflammation would slowly increase, which in turn, would be associated with reduced mitochondrial function. These two effects might be intimately related and inseparable, as they would both be a marker for system instability. The by product, of course, would be a reduced ability to capture information as suggested by the Q-SAPIENT concept. This could be predicted; in an environment with plenty of food and no need to move, less information is required to survive. But equally, anything that threatens the proton gradient, including many phenolic lipophilic plant defence compounds, would immediately induce an adaptive response.

### Does all life strive for environmental utopia?

**Figure 1:**
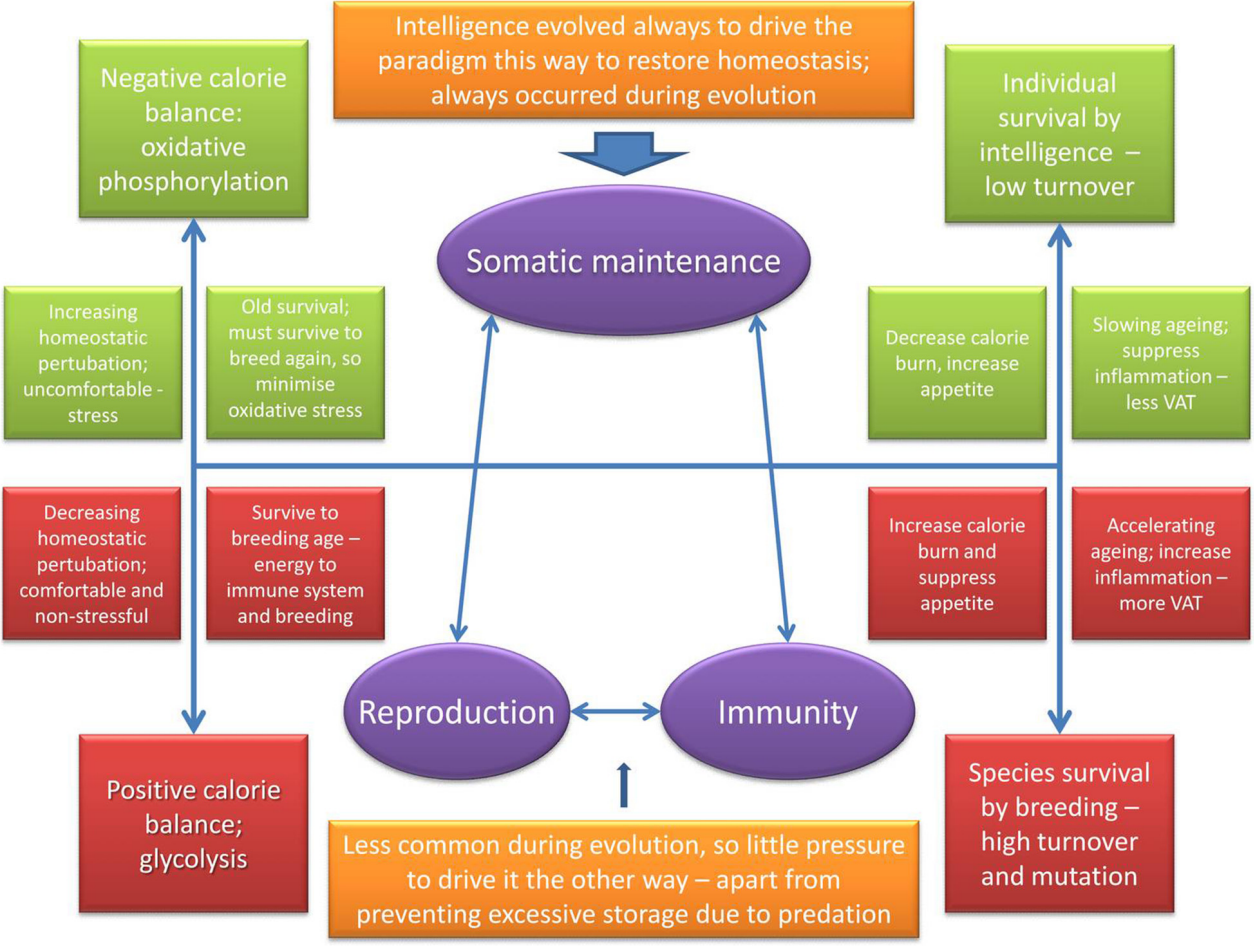
**Seeking environmental utopia.** Intelligence as a homeostatic mechanism to control environmental unpredictability.

If intelligence is a homeodynamic response to a changing environment, then it might be predicted that it will always be trying to achieve environmental homeostasis by stabilising it. In effect, it might be said that for many forms of life, the ultimate expression of intelligence would be the ability modify the environment to ensure survival. For some species, this clearly happens – for instance humans, and other species that drastically alter their environment; building shelters and “farming” are obvious examples (beavers, ants and termites, for instance). Less obvious examples might be commensal organisms, or parasites, that modulate their host’s immune systems (bacteria, for example). However, it could also be said that migration, and moving to other food sources is a simpler way of doing it. Overall, it could be that at least here on Earth, there are two mechanisms enabling species survival: the better understood Darwinian mechanism via natural selection, but also the individual adaptability driven by mild stress that stimulates (Q)-SAPIENT – and the ability to modify the environment.Thus the prediction from this is that species with larger brains would tend to live longer by displaying much greater individual adaptability, especially under mild stress, whereas shorter lived species might rely on more conventional natural selection. This would be modified by the availability of food and the ability to breed, and importantly, infection. Figure 1 summarises the idea. This is an inherently stable system, because during most of evolution, competition never really enabled any single species to achieve its environmental utopia. If it did, and in the true meaning of utopia, which is unobtainable paradise, it would expand and destroy its environment and limit its own expansion. This therefore raises the question whether any species on Earth is coming close to achieving its environmental utopia; the answer, unfortunately, is fairly obvious – we humans are not doing our planet any favours at the moment.

### All technological life might suffer an intelligence paradox – entropy’s dark laughter

The above suggests that many forms of life will not only improve their adaptability under mild stress, but also try and reduce environmental unpredictability. For most life on earth, however, this may never happen. But this may have changed with the advent of technology, as it could be argued that this is perhaps a logical step in entropy-driven evolution to accelerate energy dispersal in our universe. Technology has enabled humans to greatly increase their average energy use.

The Q-SAPIENT concept suggests that a lack of hormesis can lead to an inflammatory-based syndrome that is associated with a reduction in intelligence. Inflammation is well known to suppress brain function as part of cytokine-induced sickness behaviour, not only reducing cognitive function, but inducing depression [8,61]; this would suggest that the metabolic syndrome is associated with cognitive dysfunction, which is supported by real world data [6]. So not only is a modern environment removing a driver for intelligence, it may even be driving its decrease as populations become more overweight and sedentary. This in turn would remove the very environmental stresses that drove its evolution. In effect, humanity may be reaching this very point, a sort of evolutionary plateau of intelligence, as well as a plethora of metabolic disorders associated with the absence of hormetic factors.

**Figure 2:**
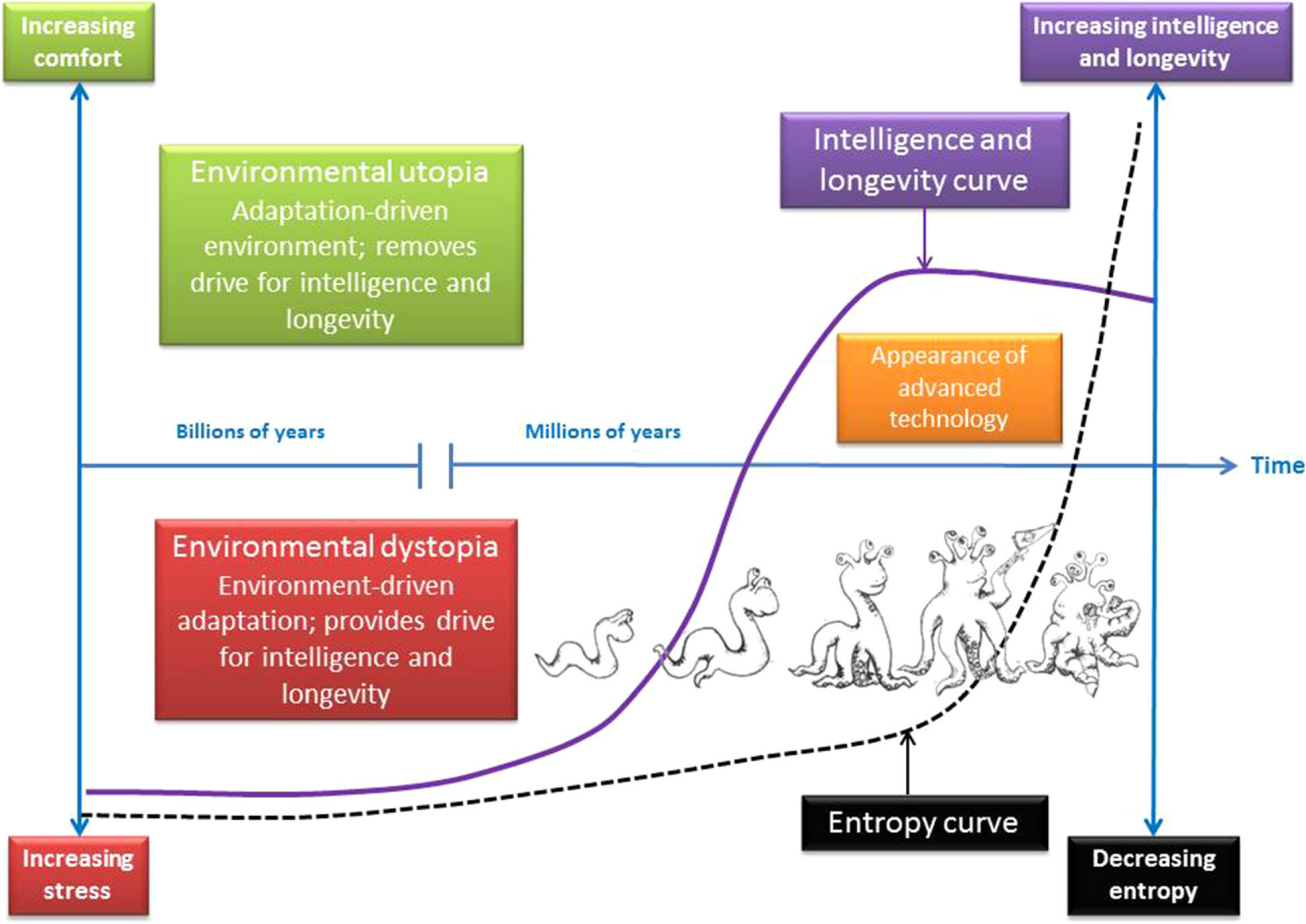
**The “intelligence paradox”: entropy’s dark laughter.** Throughout evolution the need to adapt has been driven by a stressful environment, suggesting that if intelligence ever evolved to a high enough level, it would alter the environment to remove the stress. This would thus remove the driver for further development of intelligence and adaptability (and hence longevity). However, if it reached a high enough level, it may well also fulfil the original driver for life itself; acceleration of entropy. Thus, it is possible that mankind, or ET, may be reaching a point where the original driver for entropy is still occurring through technology, but the individual driver for intelligence and adaptability has been removed. The universe could be playing a very cruel joke on us.

If hormesis has driven the evolution of intelligence, then a natural tipping point might occur when hormetic factors are suddenly removed by the advent of technology. This might suggest that in evolutionary terms all life will always naturally evolve, planetary system stability permitting, to this point. With the coming of industrial and technological ages, humans have almost complete command over the environment, which would be true from any civilization across the galaxy. In a sense, the existence of the “intelligence paradox” will be a universal and inevitable consequence of the evolution of intelligence.It could be argued that individual survival, at the expense of high population turnover and energy use, may go against the principle of life fulfilling entropy. However, this may not be the case if intelligence can increase the per capita use of energy, for instance, through technology. In effect, intelligence development reaches a point where entropy can be accelerated as technology develops. Thus technology could slow down the evolution of intelligence. So not only might a species get sicker and spend more on healthcare, its average intelligence might plateau or even fall. These are not conducive to advanced space travel. Entropy, it could be said, is making a very dark joke out of intelligence once it develops technology (Figure 2).

## Conclusions

A number of hypotheses have been developed to explain the *Fermi paradox*, ranging from self-annihilation, expense, distance, the SETI paradox, the impossibility of developing engines capable of powering star ships, to the Zoo hypothesis. While some of these questions may remain unanswered there is the possibility that as proposed by Ward and Brownlee primitive life may be very common, but advanced life is actually extremely unlikely, as most planetary systems are simply too unstable and suffer from repeated extinction events; Earth with its technological inhabitants may thus be very rare indeed [62]. However, if we assume that there are other advanced civilisations capable of building space craft out there, there may be a much more fundamental reason to explain it. At its most basic level, this may be due to an inherent property of the laws of nature whereby stress induces organisation of molecules into complex systems that harness, and use, energy to incorporate information to try and remove the very stress that enabled its creation. This eventually leads to an intelligence paradox.

### A technology paradox

One of the arguments used to explain the *Fermi paradox* is that interstellar travel is not achievable. It is possible to argue against this, indeed, it may actually an inherent property of life to fulfil entropy’s destiny to at least reach an advanced technological level. NASA is now developing technologies that could dramatically increase the speed that spacecraft could travel [5]; these could potentially greatly exceed the speed that the Voyager spacecraft are now travelling at, which is about 35,000 mile per hour, or about 0.00057% the speed of light. And it has to be remembered, although a fantastic achievement, Voyager is very old technology.

It has been estimated that in 40,000 years’ time Voyager 1 will pass within 1.2 light years of Gliese 445, an M-type sequence star, which is 17.6 light-years away (source, NASA website). The inescapable fact is that we have only been in space for 60 years or so, which is but the blink of a cosmic eye; humans went from demonstrating powered flight in 1903 to launching Sputnik in 1957. Voyager was launched in 1977, only 20 years after Sputnik. Today, we now maintain an orbiting space station and regularly send robots to most of the planets. The simple truth is that once we got in the air, we got into space very quickly. The same may well be true for any other civilisations across the galaxy. Even with the technologies on the drawing board after only 60 years in space, robots could technically be sent to neighbouring stars in the not too distant future. Interestingly, reanalysis of the Drake equation suggests a single civilisation would take either 10,000, 1,000,000 or 10,000,000 years to explore the galaxy if the colonisation front travelled at 0.1, 0.01 or 0.001% the speed of light, respectively [4]. This suggests that technology is not the issue – especially if it develops at the rate it has over the last 100 years. So what else could stop it?

### Lessons from earth; a healthcare crisis driven by environmental utopia

The proof that many human societies may well have reached their environmental utopia is all around us. Large sections of the human population now live in a sedentary obesogenic environment, with almost unlimited access to food and water, with little exposure to dramatic variations in temperature, or exposure to disease. Moreover, food has been produced to maximise calorie yield and taste, while bitterness (a property that most plant defence compounds exhibit) has been reduced. Unfortunately, these very compounds are part of the hormetic signals needed for optimal health. Indeed, much of the food that we now eat is grown in a non-stressed environment, thus reducing dramatically some of our environmental hormetic signals. “Xenohormesis” is thought to play an important role in adaptation to our environment [63] – and we have removed a lot of it.

We have previously proposed that the metabolic syndrome could be viewed as an accelerated aging syndrome induced by a lack of hormesis [12]. Another way of looking at this has been suggested by Philip Hooper and colleagues – could the metabolic syndrome be viewed as an “unconditioned syndrome” [64]? In this paper they also point out that micro-gravity, and thus space flight, is potentially diabetogenic; data which has been known about for some time [65]. Gravity is a very powerful “hormetin”, as it induces stress [66]. Hence, not only is this a good example of what happens if you remove an important source of hormesis in the environment, it also indicates that any species that attempts prolonged space flight will have to address this problem; NASA and other space agencies are continually developing ways to overcome this.

Data also suggest that as might be expected, another key hormetic factor, physical activity, is in decline on planet Earth. The latest estimates suggest that only about a third of the World’s population are doing any regular physical exercise [67]. This is reflected by the fact that a vast percentage of the population in industrialised countries is overweight or obese [68]. This is despite the overwhelming evidence of the importance of a healthy lifestyle, and attempts by many governments to improve it. Indeed, most individuals in society know what is required to achieve a healthy status, but simply do not act on it. This attitude seems to be endemic within society. For instance, a recent review found that a fair proportion of medical schools simply do not teach exercise as a separate subject, suggesting that many in the medical profession are ill-equipped to discuss, let alone implement basic guidelines [69]. This probably stems from the fact that *a lack of exercise is not seen as a risk factor* – although it is probably equivalent to smoking [70], and in all likelihood as detrimental as diets high in fat, refined sugars and low in phytochemicals. This attitude is also be reflected in a study that found that the higher the BMI of the doctor, the less likely they were to give lifestyle advice to their overweight patients [71].

This outlook probably has something to with the fact many humans are now living longer and expect, as science develops, to live longer and longer lives. Unfortunately this optimism about an ever increasing healthy life expectancy is unfounded, as the gap between the healthy and absolute life expectancy is widening all the time; in effect, morbidity is increasing and people are spending a greater proportion of their lives in ill health [72]. In fact a lot of the increase in the life expectancy in the last 100 years has probably due been to a switch in mortality from formerly untreatable infectious diseases to chronic degenerative diseases [73]; in effect, to diseases associated with the ageing process.

The truth is that these early optimistic forecasts are being revised downwards. It is now thought that the absolute maximum human lifespan is probably not far off 115–120 years, with 105 years being the most the average person might achieve, with an asymptotic limit of around 95 years [74]. However, for most developed countries in 2010, the healthy life expectancy was still closer to 70 years, with an absolute expectancy of 80 years or so [72]. In fact, there is even evidence that in some countries, such as the USA, it may actually start to fall due to obesity [75]; this is further supported by evidence that nearly 70% of the population in the USA are receiving some kind of medication [76]. Hence, it appears that the maximal lifespan of humans is fixed, as it is for most other animals, and although many people are living longer, as a species, we are not getting much closer to the theoretical limit due to our increasingly comfortable environment.

### Lessons from Earth; the cost of healthcare may prohibit space travel

In this paper we posit that there may well be a natural limiting point in evolution once intelligence can tame its environment. This point may be marked by technological development and environmental utopia, but with an increasing population that not only lives for longer, but is also unhealthier, and thus increasingly dependent on healthcare. Moreover, as the metabolic syndrome is also associated with depression and could well reduce exercise salience (the will to exercise) [77], it could also reduce the will to explore.

Healthcare costs, therefore, may well be one of the main factors why any advanced society may not go exploring beyond its own solar system. Mankind has now reached the point where over the 30% of the global population is overweight, and it is estimated that obesity is now costing between 0.7-2.8% total health care costs; but these figures probably greatly underestimate the true cost. For instance, estimates of the prevalence of the metabolic syndrome suggest that it already affects 20-30% of the population of most countries. The costs of diabetes alone are put at 12% of global health care costs [78]. But perhaps one of the most startling recent estimates is the that economic and clinical costs of obesity in the USA could be approaching $210 billion per annum [79]. This makes NASA’s budget for 2013, at $17.7 billion (source, NASA website), look paltry, especially as the GDP for the USA is more than $16 trillion (source, The World Bank).

Although very difficult to cost precisely, sending a manned mission Mars could cost, according to the Mars Society (http://www.marssociety.org), somewhere between $30 and $450 billion over 10–20 years**.** In 2007–2008, it was estimated that the obesity rate (that is people with a BMI of greater than 30 kg/m^2^), was 33.8% in the USA. If a linear trend line is used, then by 2030, this could reach 51%, although alternative logit models suggest this may only reach 42%. However, if obesity levels were to remain at 2010 levels, then savings could be approaching $550 billlion; if obesity had remained at 15%, this could be nearer $1.9 trillion [80]. Thus, by simply tackling obesity, the USA could supply NASA with enough money to send men to Mars even with the most expensive of estimates. Interestingly, George H.W. Bush advanced a plan, in 1989, to send men to Mars, but it was shelved when the estimated cost at the time hit $500 billion; had the nation already got too fat? The figures speak for themselves.

### Lessons for earth

The lessons for earth are profound. Entropy drives order by incorporating information that enables increased energy use, but this is reliant on the system constantly being stressed to maintain this order. Not only is this potentially explainable using basic evolutionary biology and biochemistry, but it might also be predicted from quantum physics. This implies that not only is life highly likely to start, but if it reaches a high technological level, it will always strive to change its environment to remove all stressors, leading to a plateau in the development of intelligence and a pandemic of metabolic diseases.

**Figure 3:**
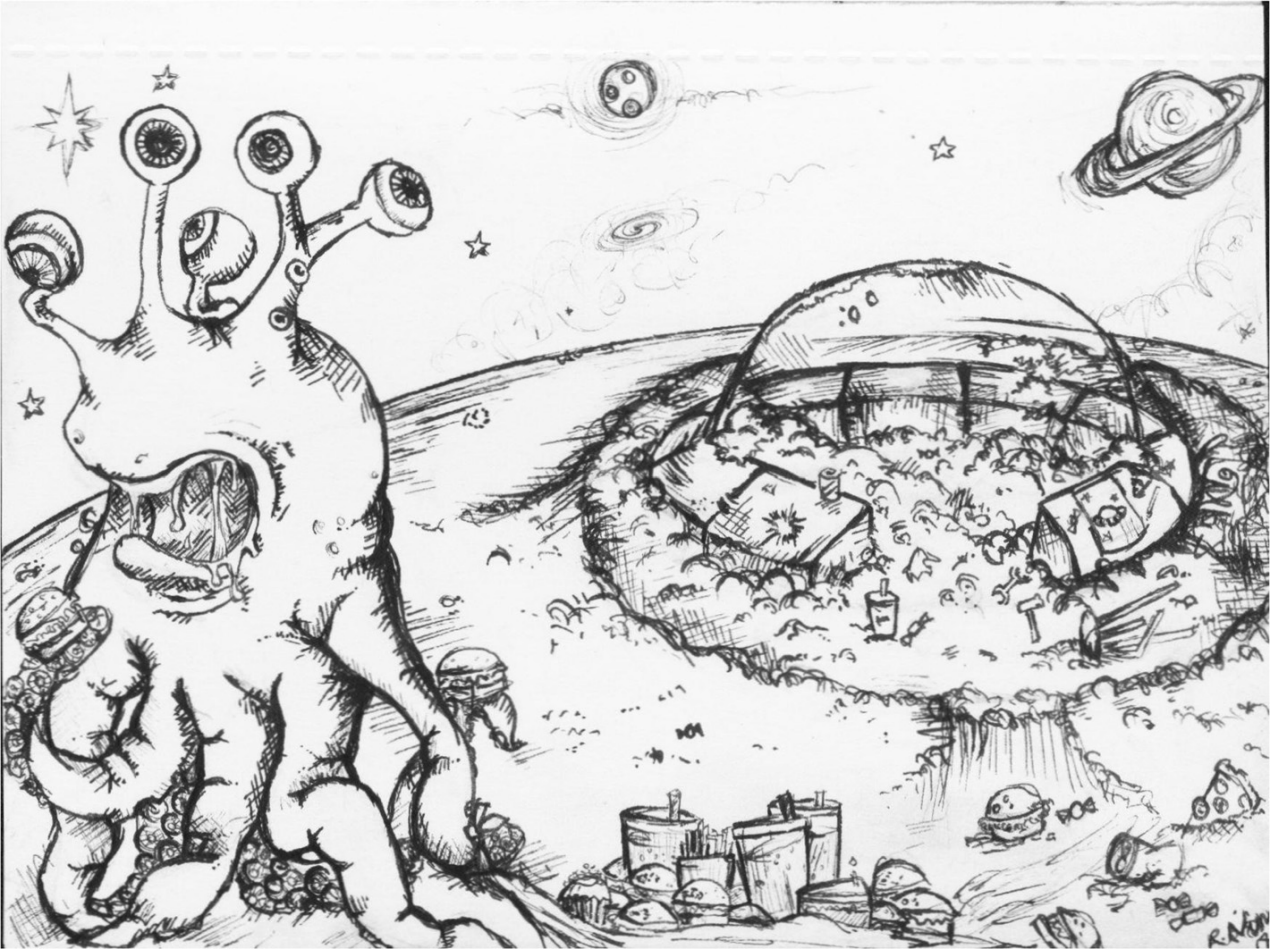
**Explaining the Fermi paradox.** Evolution of intelligence will always lead to a drive for environmental utopia. Hence, many species may well get fat and spend much of their GDP on healthcare. Life may be everywhere, but due to obesity-related medical issues, it might have to pay a healthcare tax and simply not be able to afford space travel. Without hormesis it may not even be interested.

Thus, the possibility exists that all advanced civilizations, wherever they find themselves in the galaxy, will end up with an increasing population that not only lives for longer, but is also unhealthier, and thus dependent on healthcare. This coupled with resource depletion and environmental damage could potentially lead to increasing internal conflict and societal destabilisation. All of this would reduce or halt interstellar exploration, and greatly reduce a civilisation’s sphere of potential contact. This might explain several of the theories expounded to explain the Fermi paradox. In short, ET, and maybe us, have got, or are getting, too fat for space.In summary, if we want to load the dice in our favour, then the entire population needs to live a hormetic lifestyle. Let’s hope that we, and ETs, can embrace this concept, and perhaps, one day, meet. It is a simple choice: get fit and reduce healthcare costs, and perhaps one day travel to the stars, or sit here and get fat, spending our money on healthcare and quite possibly, reducing our will and ability to explore (Figure 3). But perhaps one of the greatest challenges is not only to accept hormesis as being important on the home planet, but if we, or ET, do manage to get into space and start travelling to the stars, we would have to maintain a hormetic lifestyle on our respective interstellar space craft to ensure that we remain fit for what could be a very long journey.

## Competing interests

The authors declare that they have no competing interests.

## Authors’ contributions

AVWN was the principle originator of the intelligence paradox hypothesis and wrote and edited the manuscript. JDB contributed substantially to the discussions around the subject, editing and content. GWG provided valuable feedback on some of the topics. All authors read and approved the final manuscript.
